# Varicones and Growth Cones: Two Neurite Terminals in PC12 Cells

**DOI:** 10.1371/journal.pone.0004334

**Published:** 2009-02-02

**Authors:** Ana Mingorance-Le Meur, Alma N. Mohebiany, Timothy P. O'Connor

**Affiliations:** Department of Cellular and Physiological Sciences, University of British Columbia, Vancouver, Canada; Harvard University, United States of America

## Abstract

The rat adrenal pheochromocytoma PC12 cell line is one of the traditional models for the study of neurite outgrowth and growth cone behavior. To clarify to what extent PC12 neurite terminals can be compared to neuronal growth cones, we have analyzed their morphology and protein distribution in fixed PC12 cells by immunocytochemistry. Our results show that that PC12 cells display a special kind of neurite terminal that includes a varicosity in close association with a growth cone. This hybrid terminal, or “varicone”, is characterized by the expression of specific markers not typically present in neuronal growth cones. For example, we show that calpain-2 is a specific marker of varicones and can be detected even before the neurite develops. Our data also shows that a fraction of PC12 neurites end in regular growth cones, which we have compared to hippocampal neurites as a control. We also report the extraordinary incidence of varicones in the literature referred to as “growth cones”. In summary, we provide evidence of two different kinds of neurite terminals in PC12 cells, including a PC12-specific terminal, which implies that care must be taken when using them as a model for neuronal growth cones or neurite outgrowth.

## Introduction

The rat adrenal pheochromocytoma (PC12) cell line was originally derived in 1976 from a tumor arising from adrenal medulla chromaffin cells [1]. Since then, PC12 cells have become a very popular model for studying the signaling pathways of cell survival, proliferation and differentiation, resulting in the generation of a large amount of knowledge on these processes [2]–[5]. An interesting feature of PC12 cells, previously observed in their first description, is their capacity to grow neurite-like processes in response to nerve growth factor (NGF) [1]. The growth of neurites can also be achieved by inducing cAMP elevation and it is accompanied by other features in common with a neuronal phenotype, such as cessation of proliferation, expression of neuronal markers and secretion of neurotransmitters [3], [6]. Because PC12 cells can be passaged indefinitely and are much easier to culture and manipulate than their neuronal counterparts, they are a particularly useful model for the study of neurite outgrowth.

Neurite outgrowth is the process whereby neurons extend long processes during development to reach their targets, leading to the establishment of neuronal connections. All growing neurites are terminated at their distal end by growth cones, very motile expansions containing filopodia and lamellipodia which are capable of sampling the environment and guiding the neurite [7]. Differentiated PC12 cells, together with sympathetic and cortical neurons, are currently some of the most extensively used models for the study of neurite growth and growth cone function [8]–[10]. For example, PC12 cells were used to describe the role of vinculin in mediating growth cone attachment to the substrate [8], and also helped describe the crucial role of the Rho family of GTPases in neurite formation, outgrowth and retraction [11]–[14].

Another characteristic feature of PC12 neurites, already noted in the initial description of the cell line, is the presence of large varicosities containing catecholamines [1]. These varicosities are found in most growing PC12 neurites within 20 µm of the growth cone and might participate in the process of neurite outgrowth in these cells [9]. We noticed that often these terminal varicosities associate so closely with the growth cone that they can be easily mistaken for a part of it. Because of their high visibility, the enlarged hybrid terminals can be favored for analysis over their neighboring, less prominent, growth cones. Indeed, we detected an extraordinary incidence of this structure, composed of a varicosity in close association with a growth cone, in the literature referred to as a “growth cone”. On numerous occasions, protein enrichment at the varicosity has been reported as “growth cone enriched”, and the data extrapolated to neuronal growth cones. To clarify to what extent the neurite terminals observed in PC12 cells can be compared to neuronal growth cones, we have analyzed the different morphologies of these terminals and the distribution of growth cone markers. We found that PC12 cells display two different kinds of neurite terminals: while some neurites are tipped by proper growth cones, most neurites display the enlarged hybrid terminals that include a varicosity. This second terminal, which we have referred to as “varicone”, as opposed to growth cone, appears to be the most characteristic terminal of PC12 cells. In addition, using a varicone marker that we have identified, we have characterized the appearance of this structure back to the initial protrusions in the undifferentiated PC12 cell.

## Materials and Methods

### PC12 cell culture

PC12 cells were obtained from the American Type Culture Collection (ATCC) and cultured as described before [15]. Briefly, PC12 cells were maintained undifferentiated in Dulbecco's modified Eagle's Medium supplemented with 10% fetal bovine serum and 5% normal horse serum (Invitrogen, Carlsbad, CA, USA). To induce differentiation, cells were dissociated and plated on collagen-I (Sigma) coated coverslips in low serum conditions (1% normal horse serum) and 75 ng/ml of NGF (Invitrogen, Carlsbad, CA, USA). Once PC12 cells developed neurites, around day 5 after induction of differentiation, cells were fixed with 4% paraformaldehyde and 4% sucrose in 0.1 M phosphate buffer for 15–30 min at room temperature and processed for immunocytochemistry.

### Hippocampal neuron cell culture

Neurons were isolated from the hippocampus of CD1 mouse embryos at 16–18 days postcoitum or postnatal day 0 (Charles River, Wilmington, MA, USA) and were cultured on poly-D-lysine coated coverslips (Sigma Oakville, ON, Canada). Mouse primary neurons were maintained in Neurobasal medium supplemented with glucose, glutamine and B27 (Invitrogen, Carlsbad, CA, USA), and fixed after one day in vitro with 4% paraformaldehyde and 4% sucrose in 0.1 M phosphate buffer for 15–30 min at room temperature [16].

### Immunocytochemistry

For immunocytochemistry, fixed cells were washed in phosphate buffered saline (PBS) 0.1M and PBS-0.1% Triton X-100 and then blocked for 30 minutes in PBS-0.2% gelatin, 0.1% Triton X-100 and 2% fetal bovine serum [16]. Cells were then incubated with primary antibodies (2 hours room temperature) and secondary antibodies (45 minutes to 1 hour, room temperature) in the same solution, washing with PBS-0.1% Triton X-100 before and after incubation. Fluorescently labeled Phalloidin was included in the incubation with secondary antibodies. After this, cell nuclei were labeled with Hoechst diluted in PBS and mounted in slides with Mowiol (VWR Canlab, Mississaga, ON, Canada).

### Antibodies

The primary antibodies used where: mouse anti-βIII tubulin (Covance, Laval, QC, Canada); mouse anti-cortactin 4F11 and rabbit anti GAP-43 (Millipore, Billerica, MA, USA); goat anti-Arp3 (Santa Cruz); rabbit anti-calpain-2 (Cell Signaling Technologies, Danvers, MA, USA); mouse anti-synaptophysin (Sigma Aldrich Canada, Ontario, Canada). Alexa 488 and 546-conjugated phalloidin and Alexa 488 and 546-conjugated goat antibodies to mouse or rabbit immunoglobulin G (IgG) were from Molecular Probes (Invitrogen, Carlsbad, CA, USA).

### Image acquisition

Calpain-2 fluorescent images were obtained with an Olympus inverted confocal microscope IX81 controlled by FluoView 1000 (Olympus). For the generation of axial projections, several stacks were acquired and then projected to generate the “side views” shown in the figure. All additional images were obtained with a Zeiss Axioplan 2 Imaging microscope using a Retiga 1350EX camera (Quantitative Imaging Corporation, Surrey, BC, Canada) with Northern Eclipse software (Empix Imaging, Mississaga, ON, Canada). Negative controls in which the secondary antibody or the phalloidin were omitted were included in every experiment.

### Analysis of the literature

To survey the incidence of varicones in images in the literature and their correct identification as varicosity-containing terminals, we performed a PubMed search using the terms “growth cone PC12” and retrieved 229 articles, from which the most recent 25 that included clear pictures of PC12 terminals were used (ranging from 1994 to 2008). Two people classified each terminal as “varicone” or “growth cone” based on its morphology, and the notation used by the authors was noted. After removing those that were unclear, a total of 213 terminals were used for analysis. These included “varicones” referred to as “growth cones” or as “varicosities”, and “growth cones” referred to as “growth cones”. We found no “growth cone” misidentified. The number of articles studying neurite outgrowth or varicosities/exocytosis was also counted. We also applied the same criteria to images from our own cultures (351 terminals, 3 different experiments). Data was represented as % of total.

## Results

### Morphological characterization of PC12 growth cones and varicones

**Figure 1:**
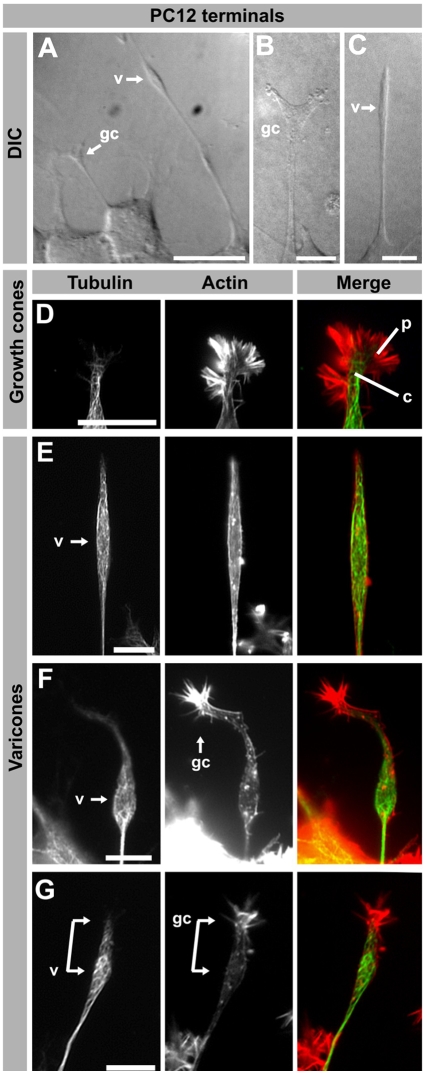
Morphological characterization of PC12 varicones and growth cones. (A–C) Examples of differentiated PC12 cells observed using Differential Interference Contrast (DIC). A large varicosity at the tip of many neurites is apparent (“v” in A and C) while other terminals resemble neuronal growth cones (“gc” in A and B). (D–G) Fluorescence images of several PC12 cell neurite terminals labeled with a β-III-tubulin antibody and phalloidin to visualize tubulin and actin. While some terminals display a morphology similar to neuronal growth cones, including a tubulin-rich central domain (c) surrounded by an actin-rich peripheral domain (p) as shown in D, others have a large varicosity (E–G) and “growth cone” that can be collapsed (E) or more clearly visible (F–G). In some neurites, both the varicosity (v) and the growth cone (gc) appear as a connected structure (connected arrows in E). Scale bar A = 20 µm, B–G = 10 µm.

PC12 cells grown in the presence of NGF have a polygonal shape and extend processes of different morphologies (Figure 1). Using Differential Interference Contrast (DIC) microscopy, the presence of numerous big and elongated varicosities was very noticeable (Figure 1A–C, “v”), as these appeared extraordinarily thick in comparison to the rest of the cell processes (Figure 1A–B, “gc”). Because these structures were not present in every neurite, and seemed to vary in shape, we resolved to analyze the different morphologies of PC12 neurite terminals by looking at the distribution of tubulin and actin within fixed cells. We found that PC12 cells had two kinds of terminals, based on their cytoskeletal composition and morphology. First, some neurites of PC12 cells have ends that closely resemble neuronal growth cones, with a neurite shaft and growth cone central domain rich in tubulin surrounded by actin-rich lamellipodia and filopodia (Figure 1D). A second kind was characterized by the presence of a large varicosity very near the neurite tip and a more or less visible growth cone (Figure 1E–G). The varicosities were surrounded by a net of microtubules, but did not contain actin-rich structures, which are also properties of the neurite shaft (Figure 1E). On occasions it was easy to identify both the varicosity and an associated growth cone (Figure 1F, compare to Figure 1 in [9]), although they might also appear as a connected structure (Figure 1G). In other instances, the existence of a growth cone was not evident and a varicosity seemed to be the only terminal structure, but the frequent presence of filopodia at the tip of these varicosities suggested the existence of a growth cone that had retracted or collapsed just prior to fixation. We refer to the special terminal, characterized by having a varicosity in addition to a growth cone (that could be collapsed or not), as a *varicone*. Because varicones were frequently observed in PC12 cells but not neurons, we further characterized their molecular nature in order to have a better understanding of the contributions of varicosities and growth cones to a varicone.

### Immunocytochemical characterization of PC12 growth cones and varicones

**Figure 2:**
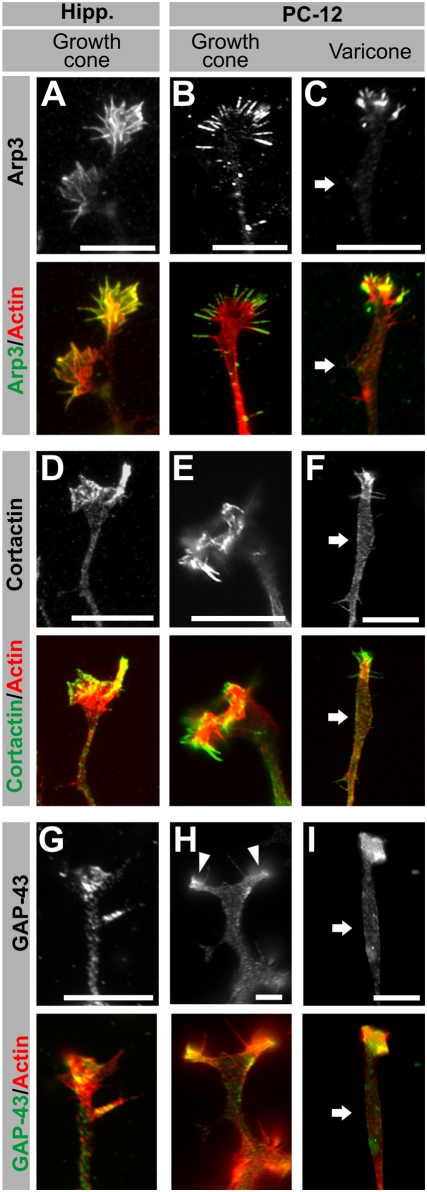
Immunocytochemical characterization of PC12 terminals I: growth cone markers. (A–I) Fluorescence images of hippocampal neuron growth cones, PC12 growth cones and PC12 varicones, immunolabeled with growth cone markers (white or green) and co-labeled with phalloidin (actin, red). Arp3 (A–C), Cortactin (D–F) and GAP-43 (G–I) localize to the actin-rich regions of the growth cones, including the actin-rich region associated with varicones (C, F, I), but not to the varicosity associated with varicones (white arrow). Scale bar = 10 µm.

In order to discern those areas of PC12 terminals that more closely resemble neuronal growth cones, we examined the localization of typical growth cone markers by immunocytochemistry in fixed hippocampal neurons and differentiated PC12 cells (Figure 2).

Arp3 is a member of the Arp2/3 complex and participates in actin filament branching by creating new branching sites [17]. In neurons, Arp3 labeling was restricted to the growth cone (Figure 2A), where it colocalized with F-actin, consistent with previous reports [18]–[20]. Similarly, those terminals of PC12 cells that morphologically resembled growth cones had a clear enrichment of Arp3 at their terminal filopodia and lamellipodia, while there was no labeling along the shaft (Figure 2B). This pattern, restricted to the growth cone, was also conserved in neurites ending in varicones (Figure 2C). Thus, Arp3 labeling was restricted to the very end of the neurite, where actin staining indicated the presence of a growth cone, while it was absent from the shaft, including the varicosity (arrow in Figure 2C). These results were supported by the expression pattern of two additional growth cone markers: the actin-associated protein cortactin [18], [21], and the membrane protein GAP-43 [22], [23]. We found that cortactin was enriched at the growth cone in hippocampal neurons (Figure 2D) and in PC12 cells (E), including the growth cone compartment of varicones (Figure 2F). PC12 cells varicosities, however, displayed little labeling, which is similar to the pattern observed in the rest of the shaft (arrows in Figure 2F). The same pattern was reproduced with GAP-43 (Figure 2G–I). Together, these results indicate that despite being frequently morphologically merged, the growth cone component of varicones is restricted to the actin-rich region and does not extend into the varicosity component.

**Figure 3:**
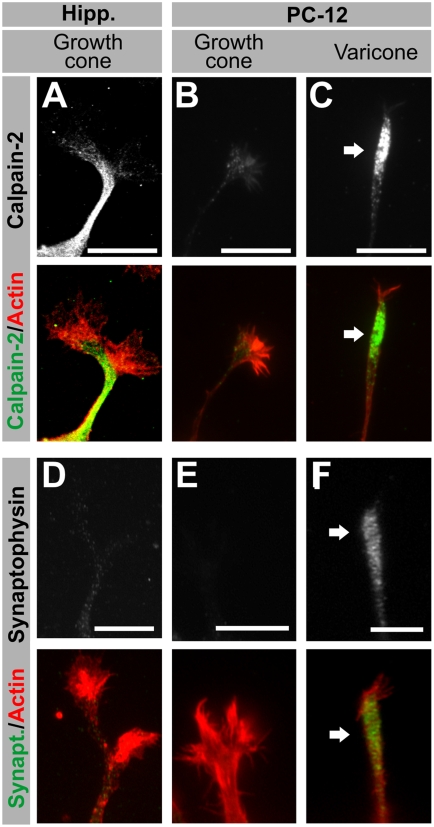
Immunocytochemical characterization of PC12 terminals II: varicosity markers. (A–I) Fluorescence images of hippocampal neuron growth cones, PC12 growth cones and PC12 varicones, immunolabeled with non-growth cone markers (white/green) and co-labeled with phalloidin (actin, red). Calpain (A–C) is a protease enriched at the neurite shaft in neurons (A) but not so in PC12 cells (B–C), where it localizes almost exclusively to varicosities (white arrow). Synaptophysin (D–F) is a presynaptic marker that is barely detected in young growing neurites in hippocampal neurons (D) but enriched at PC12 varicosities (arrow in F). Scale bar = 10 µm.

We then used a different set of markers to assess whether the varicosity in PC12 varicones partially invades the apparent growth cone, therefore generating a combined area, or whether both components remain distinct despite their close apposition. In our previous work, we had identified calpain-2 as a protein enriched in neurite shafts, and therefore a good marker for this purpose [18]. In hippocampal neurons, calpain-2 immunolabeling localized to the neurite shaft, while it was very low in the growth cone actin-rich areas (Figure 3A). In contrast, calpain-2 labeling was very low along PC12 neurites, and it had a clear vesicular pattern (Figure 3B–C). In PC12 neurites ending in growth cones, calpain-2 was particularly absent from the actin-rich region (Figure 3B), supporting our previous observations with growth cone markers. Surprisingly, calpain-2 accumulated in PC12 varicosities at varicones (arrow in Figure 3C). This data indicates that the varicosity is characterized by a distinct molecular identity in this region of the terminal.

**Figure 4:**
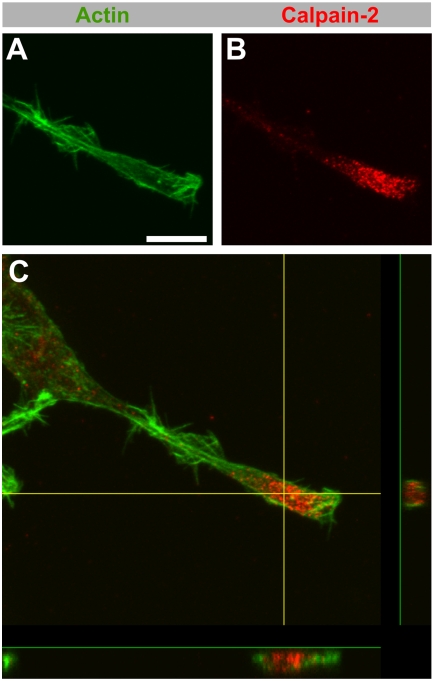
Calpain-2 subcellular localization in PC12 cells. (A–C) Double immunolabeling of calpain-2 (red) and actin (phalloidin, green) in a differentiated PC12 cell. Calpain-2 (B) localizes almost exclusively to the varicosity contained in the varicone (A). In a Z-projection across the varicone (C), actin staining is detected around the varicosity, while calpain-2 fills the space within. Scale bar = 10 µm.

Next, because PC12 varicosities have been described to be similar to presynaptic terminals [24], we explored the localization of synaptophysin a synaptic marker in PC12 terminals. Synaptophysin, a presynaptic marker, is absent from neuronal growth cones at the time they are extending (Figure 3D), and it was also absent from PC12 growth cones (Figure 3E). It was, however, enriched in varicones, where it was limited to the varicosity portion (Figure 3F). We could indeed confirm using z-stacks that varicosity markers fill the entire volume of the varicosity without invading the growth cone component (Figure 4). Therefore, despite being morphologically merged, the varicosity component of varicones does not extend into the growth cone compartment.

### Calpain-2 pattern in growing PC12 processes

**Figure 5:**
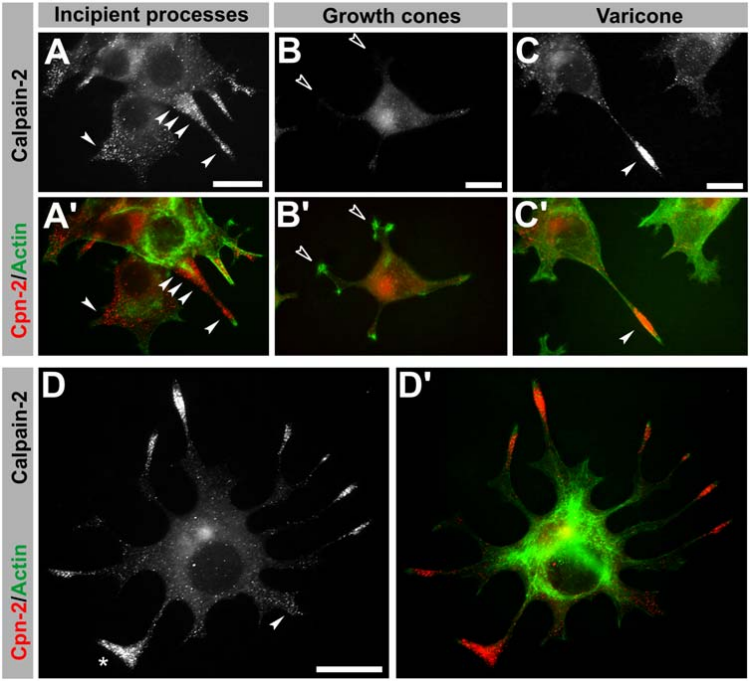
Onset of PC12 process outgrowth. (A–C) Double immunolabeling of calpain-2 (white or red) and actin (phalloidin, green) in PC12 cells at different stages of differentiation. (A) Calpain-2 labeling in the initial stages of process outgrowth of PC12 cells concentrates around and along the initial buds (arrowheads). (B) Some neurites don't have visible accumulations of calpain-2 (empty arrowheads), and they invariably lack a varicosity (as determined by morphology). (C) Some neurites develop a clear varicone (arrowhead) and calpain-2 labeling concentrates at the varicosity. (D) An example of PC12 cell showing multiple processes with a variety of morphologies. Calpain staining uncovers the varicosity contained in the PC12 terminals, including those in which the presence of a varicosity was not easy to determine by morphology, such as those of triangular shape (asterisk). An incipient process is also highlighted by the presence of calpain-2 puncta (arrowhead in D). Scale bar = 10 µm.

Given the differences in the morphology of varicosities and their association with other structures, it is not clear where they are generated and whether they are present in neurites where an enlarged terminal is not apparent. Therefore, we took advantage of the restricted distribution of calpain-2 to varicosities to track their generation in undifferentiated PC12 cells (Figure 5). Soon after NGF addition to PC12 cells, calpain-2 puncta tended to accumulate in the incipient processes (arrowheads in Figure 5A), not only at the tip but also in the nascent area, suggesting they are being transported. Once neurites were visible, those with clear growth cones had very low levels of calpain-2 along their processes (open arrowheads, Figure 5B) while cones, containing varicosities, concentrated the greatest amount of the protease (arrowhead in Figure 5C). Although the pattern varied between cell and even between individual processes, calpain-2 was consistently enriched in the incipient sprouts and populated the vast majority of neurite tips. In fact, detection of terminals without a visible varicosity or calpain-2 labeling was an exception. In the example shown in Figure 5D, all neurites display very visible varicones with the varicosity containing large amounts of calpain-2, including the frequently observed triangular-shaped terminals (asterisk in Figure 5D). Similarly, those neurites that are starting to develop (arrowhead) already show enrichment in calpain-2. Thus, varicosities are intimately related to neurite growth in PC12 cells, being present from the onset of process initiation, and staying closely associated with the growth cone to the extent of creating a typical PC12-specific hybrid terminal.

### PC12 cones are frequently misidentified and overrepresented in the literature

**Figure 6:**
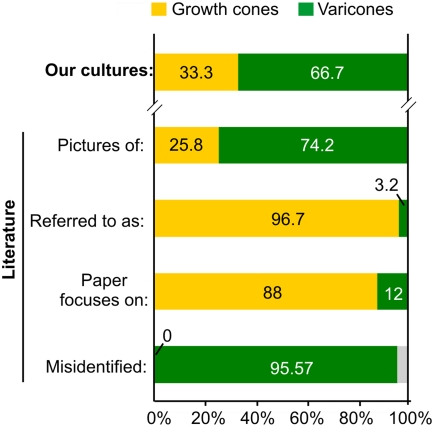
Misidentification of **varicones** in the literature. Histogram showing the frequencies of growth cones and varicones in our cultures (top bar) and in the images published in the literature (second bar), including the notation used in those articles (third), the central topic of the article (fourth) and the frequency of errors in the identification of growth cones (always correctly identified) or varicones (usually regarded as a growth cone) in the literature (lower bar).

One of the main reasons we undertook this study was the large number of PC12 varicosities we saw identified as growth cones in the literature. As an attempt to measure the incidence of this representation, we surveyed the most recent articles describing neurite outgrowth and protein localization in PC12 neurites that showed clear pictures of PC12 “growth cones” (Figure 6). Using morphological criteria, we classified each terminal as a growth cone or a cone and compared it to the identification given by the authors. In our own cultures, varicones were present in approximately two thirds of total neurites (234/351), while growth cones accounted for the remaining third (117/351, Figure 6). These proportions were not altered during the time in vitro, at least during the first week after induction of a neuronal phenotype with NGF (not shown). The number of each terminal we could identify in the images present in the PC12 literature were slightly biased towards an overrepresentation of varicones (158/213 terminals), probably owing to their high visibility. Strikingly, while three quarters of the images corresponded to PC12 varicones, virtually all of them were referred to as growth cones (206/213, Figure 6), with a few exceptions correctly identified as varicosities. In fact, all growth cones were correctly identified, while 100% of misidentifications corresponded to varicones being thought to be growth cones (Figure 6). Because the articles studying growth cones in PC12 cells (22/25) were much more numerous than those that addressed the function of varicosities (3/22), this suggests that the impact and consequences of this misrepresentation is greatest in the field of neurite growth.

## Discussion

**Figure 7:**
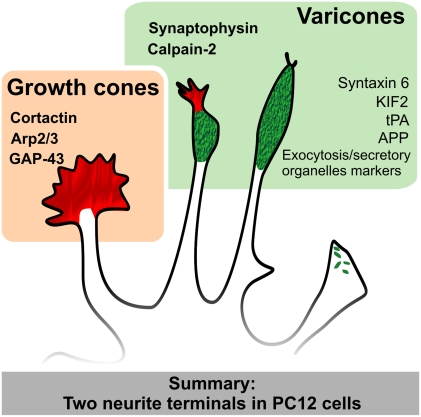
Summary of the findings. PC12 terminal morphology include the genuine growth cone (red, left) and two examples of varicones containing a varicosity (green) and a more or less apparent growth cone (green/red or green). Both terminals are characterized by the expression of different set of markers (those shown in this article are in bold).

PC12 cells are one of the traditional models for the study of neurite outgrowth and growth cone behavior. Our results show that that PC12 cells display a special kind of neurite terminal that includes a varicosity in close association with a growth cone. This special terminal, or varicone, is characterized by the expression of specific markers not present in growth cones and can be tracked back to the incipient neurite buds in the PC12 cell (Figure 7). Our results suggest that investigators should be cautious when extrapolating data obtained in PC12 as models for neuronal growth cones.

### PC12 cells generate two different terminals

The presence of large varicosities in PC12 cells is well known [1], [6], [25]–[27]. These varicosities share features with presynaptic terminals [24], [28] and contain large numbers of catecholaminergic vesicles, which can be secreted when stimulated [6], [29], [30]. We noted however, that varicosities tended to locate to the very end of the neurite (within 10 µm of the tip) giving the impression of being unusually large growth cones. Our results indicate that varicosities associate very closely with PC12 growth cones, to the extent of possibly being involved in the neurite outgrowth process. The involvement of both the varicosity and the growth cone in neurite advance is also suggested by time lapse studies of growing PC12 neurites ([9], see also http://neurite.embl.de/html/movies.html). Thus, the varicosity advances together with the growth cone and stays at the tip of the neurite even when the growth cone temporarily retracts or collapses. In this circumstance, processes would appear in fixed cells as a neurite with a terminal varicosity and no visible growth cone (For example Figure 1C, E), which would correspond to a particular state of the hybrid terminals. Because this hybrid terminal of varicosity and growth cone is very frequently observed in PC12 cells, but not in neurons, we suggest the different notation of “varicone” for it, while keeping the term growth cone for those terminals without an associated varicosity.

It is interesting that PC12 cells have two kinds of neurite terminals. Is there any difference or advantage for a neurite to have a growth cone versus a varicone? Aletta and Greene noted that neurites that have varicones (“a varicosity within 20 µm of the growth cone”) elongate with much higher frequency than those with growth cones [9]. In their video recordings, over 90% of neurites with varicones were extending, while more than half of those that only had a growth cone were rather static [9]. They also observed that the loss of a varicosity was typically accompanied by a cease of neurite outgrowth [9], supporting a role in neurite outgrowth. In agreement with this, neurites with varicones in our study were typically longer than those that had only growth cones (see for example Figure 1A). While it could be suggested that larger neurites may have acquired varicosities after a significant amount of growth, we have noted that calpain-2-positive vesicles contained in the varicone migrate into the neurite from the time the initial bud sprouts, arguing against this hypothesis. The fact that PC12 cells use a hybrid terminal for neurite elongation makes them remarkably different to neurons, which questions their utility as models of neuronal growth cones.

We have also established that despite being morphologically merged and being both involved in neurite outgrowth, the varicosity and the growth cone components of the varicone remain segregated. This means that the contents of the growth cone are restricted to the actin-rich region and do not extend into the varicosity. Reciprocally, we have shown that the varicosity component of varicones does not extend into the growth cone, as many proteins selectively localize to the varicosity. A remarkable example of this latter case is calpain-2. Calpain-2 is a protease that we have previously shown controls neurite shaft consolidation by degrading the machinery needed to promote sprouting [18]. Therefore, if calpain-2 localized to, or was active at the growth cone, it would lead to growth cone collapse by degradation of those same proteins that are needed to generate filopodia and lamellipodia [18], [31]–[33]. In agreement with this, the surprising concentration of calpain-2 in PC12 cones was restricted to the varicosity, which would prevent it from interfeering with growth cone function. Interestingly, calpain inhibitors promote neurite elongation in PC12 cells [34], and both NGF or cAMP reduce calpain activity in these cells [35]. Because several calpain members are involved in vesicle trafficking and exocytosis [36], and because the calpain-2 pattern in PC12 cells is particularly vesicular, these results suggest that the vesicles contained in the varicosity support the growth cone function and therefore contribute to the growth of the neurite.

### PC12 varicones and their impact to neurobiology

The finding that three quarters of the recent studies done with PC12 growth cones actually examine varicones, and in particular the proteins that localize to the varicosity component, is surprising. Undoubtedly these findings have influenced the current understanding of protein function and growth cone dynamics. In some cases, proteins had been described as enriched in growth cones while images showed unambiguous and specific location to the varicosity in the varicone [37]–[41]. In fact, some of these images contained a neighboring growth cone clearly devoid of the protein located to the varicosity [41]. Based on these reports, we can add to our list of varicone markers the proteins syntaxin 6, KIF2, tPA or even APP ([38]–[40], Figure 7). In other occasions growth cones are correctly identified, and some articles are exemplary (see ref [42] with more than 10 growth cones). While we cannot measure the ultimate impact of this misidentification in our understanding of growth cones, it has clearly led to a mischaracterization of these proteins and conclusions regarding their role in neurite outgrowth. In the absence of corroboration using primary neurons, this is likely to have contributed to the contradictory reports that we often see in neuronal cell biology.

### PC12 cells as a model for neuronal growth cones

In the light of our results, one wonders whether PC12 cells are a reliable model for the study of neuronal growth cones. Based on their morphology and expression of specific markers, it is clear that some PC12 cell neurites have individual growth cones that closely resemble those of neurons. This is particularly evident in the case of Arp3, cortactin and GAP-43, which we have shown to have the same growth cone localization in PC12 cells that they have in neurons [18]–[20], [22]. Indeed, some studies have succeeded in identifying growth cones accurately when describing protein localization at neurite terminals [42], and we urge researchers to follow the same criteria in their studies, and whenever possible, use varicone or growth cone markers as a control. In the absence of these controls or validation using primary neurons, expression data cannot be extrapolated to neuronal growth cones in a reliable way.

A more complicated issue is using PC12 cells as a model for neurite outgrowth. Given the correlation between varicosities and neurite elongation in PC12 cells, together with the much higher incidence of varicones over growth cones, pharmacological and genetic studies aimed at assessing the involvement of a protein in neurite outgrowth should be interpreted carefully. Manipulations that target the varicosity are likely to interfere with varicone functioning and therefore with neurite elongation in PC12 cells, producing data difficult to interpret.

In summary, we have provided evidence of two different kinds of neurite terminals in PC12 cells. In particular, we have characterized the structure of varicones, a PC12-specific variation of neuronal growth cones that incorporates a varicosity. While the advantages of using a cell line and the usefulness of controlling PC12 cell differentiation make these cells easy and practical to work with, care must be taken when using them as a model for neuronal growth cones or neurite outgrowth.
